# Keep them coming

**DOI:** 10.1093/jhps/hny052

**Published:** 2019-01-11

**Authors:** Richard Ricky Villar

**Affiliations:** Editor-in-Chief, Journal of Hip Preservation Surgery

For the past 6 months I have been learning to be a journalist, a future chosen path, as I have reached a certain age. For sure it has been interesting training, but the similarities between the world outside, and the scientific bubble of academic publishing that *JHPS* inhabits, are at times frightening.

Freelance text journalists will have a lower chance of an unsolicited submission being published than any scientific author, and they will be paid a pittance for their efforts. Few have retired wealthy from writing. Publishers are going to the wall more frequently than is comfortable, while the internet is making significant inroads to their profits. Advertising is harder to find—many publications rely on it—which means standards can be allowed to lapse and articles can be commissioned, or accepted, simply to ensure that advertising income follows.

Take a look at the next travel article you read in a mainstream publication and decide for yourself if you think it has been written with advertising in mind, or for a genuine desire to tell an original story. Faced with these frustrations, self-publishing, now called independent publishing, has taken off remarkably. It is now the way of things. Right now, >50% of new e-books are independent, a number that is set to increase further. No wonder the larger publishers are worrying.

There is a problem, too, as the headlong dash for independent publishing runs the risk of compromising quality. Whole conferences are dedicated to being noticed, how to sell, how to feature on social media. Not once have I attended a meeting, in the great wide world of writing, and I attend plenty of them, where anyone has even mentioned elegance of the prose.

Enter at this point, academic publishing. The subscription journals are struggling because advertising is harder to find, so they are forced to think continually how best to ensure their survival. No longer are readers loyal. We are like sheep in a field, grazing here and there. We take a piece of information from one journal, another from somewhere different, we then undertake an internet search and might also look at social media. This is the way it is done these days. No longer do we trust one journal alone and rely on it to offer everything we need, both as a source of information, but also as an outlet for our research. We look around and shop around. It is simply the way we do it.

If subscription journals are struggling, then so is Open Access, as its simplicity allows so many players to compete. I am certain *JHPS* readers will receive regular requests to write for journals based in far-flung lands, or to present at conferences that seem to have no focus. Some of these invitations are a privilege to receive and are unquestionably above board and valid. Others are more dubious. I sometimes ask myself the question, is this journal, is this conference, after my money or my expertise? Sadly, and all too often, it can be the former. Money, not expertise. But their existence dilutes an already overstuffed market.

This makes it hard for any credible journal to compete, be it subscription, Open Access, or a combination. There is a temptation to lower standards, increase acceptance rates, select papers that may encourage advertising—all manner of tricks to keep a journal afloat. It is the role of the Editor, Deputy Editors and Editorial Board to ensure this does not happen. At *JHPS* we are blessed by incredible editorial support, without which we simply could not function. I realize that I acknowledge, and appreciate, its role repeatedly. That is intentional.

But top of the list for our journal’s success must be the authors and, of course, our readers who cite what we publish. Right now, we are doing brilliantly, and I am grateful for your support. But we need to keep it that way in the face of challenges from many sectors. We need to be sure that *JHPS* remains in the forefront of hip preservation surgery. It reached that point in record time. We need it to stay there in perpetuity. Without authors submitting, and submitting decent work, a journal will ultimately fail. I rely on us all to be sure that does not happen to our journal.

When it comes to improving your chances of being accepted, there are two simple rules I would encourage. They apply as much to work in the wider world of writing, as they do to *JHPS*.

First, have a clear message. Be specific. If you cannot summarize your work in a single sentence, the chances are your submission will be too vague. For example, do not say something to your trainee like, ‘Let’s have a look at my last 50 osteotomies’. Make it clearer, such as, ‘Let’s have a look at the rehabilitation of my last 50 osteotomies’. Be as focussed as you can.

Second, and as a writer the most important, never submit your first effort. Finish your work, at least think you have finished, but at that moment do not click ‘Send’. Wait a few days, preferably a fortnight, and revisit what you have done. I wager you will think your first effort was futile and you will edit and edit again. This editorial, for example, has been edited multiple times. Even so, I feel I could have done better. Remember also, Thomas Jefferson. It was he who said, ‘The most valuable of all talents is that of never using two words when one will do’. There is always room for making things shorter.

So, keep ‘em coming! Please think of *JHPS* first, last, and continually.

Turning more specifically to our journal. I thought our last issue of *JHPS*, issue 5.3, was splendid. Yet again I was spoiled for choice. I was especially interested to read the two review articles on venous thromboembolism associated with hip preservation surgery, one by Rezaie, Azboy and Parvizi [1] where a small twice-daily dose of aspirin was sufficient to reduce an already small risk. Meanwhile Bolia *et al*. [2] agreed that the risk of VTE was small after hip arthroscopic surgery but suggested that prophylactic measures should be decided on a case-by-case basis. Either way, I was pleased to learn that the risk of VTE was small.

And for this issue, our latest, issue 5.4, which papers especially appeal? All of them of course, otherwise they would not have been accepted, although two in particular stand out. There is that by Krueger *et al*. [3] on injectable autologous chondrocyte implantation, in which 32 patients were followed for 3 years and demonstrated significant hip score improvement, despite the presence of large acetabular defects. That is truly astonishing. The other was a simple question of patient expectation. What do patients feel about their results after periacetabular osteotomy when compared with their surgeon? We have Boye *et al*.[4] to thank for that. Unsurprisingly, and in keeping with similar studies in so many other specialties, there was a frequent discrepancy between patient and surgeon expectations, with patients being more optimistic than their surgeons.

So, as ever, please enjoy this issue of *JHPS*. It is published for you, the hip preservation practitioner, and is filled from cover to cover with brilliance. I commend this issue to you in its entirety.

My very best wishes to you all.
